# Correlates of condom use in a sample of MSM in Ecuador

**DOI:** 10.1186/1471-2458-6-152

**Published:** 2006-06-12

**Authors:** Juan-Pablo Gutiérrez, Diana Molina-Yepez, Ken Morrison, Fiona Samuels, Stefano M Bertozzi

**Affiliations:** 1 Division of Health Economics and Evaluation, National Institute of Public Health, Cuernavaca, Mexico; 2 Juan César García Institute, Quito, Ecuador; 3 POLICY Project, Mexico; 4 Research and Evaluation Unit, International HIV/AIDS Alliance, Brighton, UK; 5 Center for Economic Research and Education, A.C. (CIDE), México DF, Mexico

## Abstract

**Background:**

In Ecuador, the prevalence of HIV in the general population is approximately 0.3%. However, up to 17% prevalence has been reported among specific groups of homosexual and bisexual men. The objective of this study is to explore correlates of condom use among men who have sex with men (MSM) across eight cities in Ecuador.

**Methods:**

A cross-sectional survey design was used. A questionnaire including variables on sexual behaviour, demographics, and socio-economic characteristics was distributed to a sample of MSM in eight Ecuadorian cities.

**Results:**

Information was obtained for 2,594 MSM across the eight cities. The largest subcategory of self-identification was active bisexuals (35%), followed by those who described themselves as "hombrados" (masculine gays, 22%). The mean age was 25 years, and the majority were unmarried (78%), with a median of 10 years of schooling (IQR 7 – 12). Regarding condom use, 55% of those interviewed had unprotected penetrative sex with each of their last three partners, and almost 25% had never used a condom. The most important correlates of condom use were single status, high life-skills rating, and high socio-economic status (RP 5.45, 95% CI 4.26 – 6.37; RP 1.84, 95% CI 1.79 – 1.86, and RP 1.20, 95% CI 1.01 – 1.31, respectively).

**Conclusion:**

Our data illustrate the urgent need for targeted HIV-prevention programs for MSM populations in Ecuador. MSM have the highest HIV prevalence in the country, and condom use is extremely low. It is imperative that prevention strategies be re-evaluated and re-prioritized to more effectively respond to the Ecuadorian epidemic.

## Background

In Latin America, the predominant group of individuals with HIV/AIDS is men who have sex with men (MSM). Despite the feminization of this epidemic in several Latin American countries in recent years, the highest infection rates remain among MSM, both in the gay community as well as among men who do not self-identify as homosexual or bisexual, but engage in penetrative sex with male partners [1].

Currently, there is a dearth of information on risk behaviours among MSM in Ecuador, and few explanations of why the epidemic remains so confined to this population. Given the evidence of high prevalence of bisexual behaviour, authors have postulated that the bisexual population could function as a bridge to the heterosexual population [2-5]. Comprehensive studies exploring the spread of HIV/AIDS in Ecuador do not exist. We know that transmission is sexual, concentrated in MSM, and there are important potential gateways between MSM and other subpopulations. For instance, in Catholic Latin American societies, many MSM are married men in heterosexual relationships who have extramarital homosexual sex [6-8].

The available data point to high levels of HIV infection in MSM in Ecuador. A sero-survey performed between 1999 and 2002 with 632 MSM in Ecuador as part of a regional project revealed an HIV-1 seroprevalence of 17%, the fourth highest among the countries studied so far. A seroprevalence of 23% was observed for MSM aged 25 to 29 years. Factors associated with infection included more sexual partners and a history of sexually transmitted infections (STI) [9].

To illustrate the lack of publications relating to MSM in Ecuador, searches for "Ecuador" and "MSM, homosexual, or gay", in the U.S. National Library of Medicine yielded only two references, which are both cited in this report, and 12 documents from AEGIS (AIDS Education Global Information System) [10], including conference abstracts, reports, and press information. Of these, only 4 involved actual studies (the rest being newspaper or magazine articles), and these were based on data already discussed here.

Given the epidemiological situation and lack of data on sexual behaviour in this group, which plays such a central role in the dynamics of the regional epidemic, it is vital to obtain more population-specific information to inform decisions on policy, programming, and intervention design. Significant factors in the context of many developing countries include the relative lack of a gay identity, as well as antagonistic attitudes towards same-sex sexual practices. Therefore, successful prevention campaigns should consider particular strategies that will effectively target MSM in a context of discrimination and marginalization.

The objective of this report is to present information from a survey of MSM behaviour in eight cities in Ecuador. The survey was implemented in the context of evaluating the Frontiers Prevention Project (FPP), an HIV prevention initiative focused on key populations that was designed with the active participation of the target populations.

## Methods

We performed a survey of MSM and SW in eight Ecuadorian cities (Machala, Milagro, Daule, Esmeraldas, Santo Domingo, Quevedo, Quito & Guayaquil). These cities were selected on the limited data available on STI and HIV prevalence as well as the views of Ecuadorian NGOs working with MSM and AIDS regarding the distribution/concentration of MSM risk behaviour among large and medium-sized cities.

On one hand, the baseline survey aimed to inform the design and implementation plan for prevention activities within the FPP. On the other hand it established a point of comparison to allow the measurement of whether, and to what extent, the project has an impact, as compared to the changes that occur over the course of the project in the absence of FPP interventions. The overall framework of the evaluation is broader than the scope of this paper; the methodology described here will be limited to the aspects relevant to the analysis presented below.

The study was approved by the Institutional Review Boards (IRB) of the National Institute for Public Health in Mexico (INSP), the National Health Council in Ecuador, and the International HIV/AIDS Alliance in the United Kingdom.

### Target population and sampling

The target population was defined as MSM within feasible reach of prevention activities. Feasibility was related to meeting place attendance. In order to estimate the size of the target populations, a mapping exercise was undertaken in each city. Members of the populations under survey visited the selected cities and, with the help of the local population and other key informants, mapped the sites where MSM were concentrated, while simultaneously providing numerical estimates of the populations in each of these areas.

Total sample size was estimated, based on the expected differences in condom use rate at the end of the project, and adjusting for the design effect of cluster sampling, since the unit of randomization was cities.

Results of the mapping were applied to assign sample size by city, although it should be highlighted that mapping data primarily disclosed numbers of easily accessible MSM, that is, individuals frequenting common meeting spots for these groups.

### Data collection

The questionnaire was developed by an international multidisciplinary and inter-institutional team. The design process was coordinated by the lead researcher responsible for the FPP evaluation, and included an active collaboration of local researchers, as well as key local stakeholders, particularly members of the populations under survey.

This questionnaire was designed to provide a comprehensive image of MSM and their contexts, including socio-demographic information (age, education, work situation, assets), data on sexual behaviour (particularly, types of sexual partners, kinds of sexual practice, condom use, and other details of their last three sexual partners), information on existing regular partners, knowledge about HIV/AIDS and STDs in general, and attitudes towards people living with HIV.

To ensure suitability of language and context, a series of focus group discussions and in-depth interviews with MSM were performed. Data collection took place between July and September 2003. Anonymous face-to-face interviews were conducted with participants. Most interviews took place in locations frequented by MSM when away from work, while others were conducted in work places. Since names were not registered, data cannot be linked to specific individuals. Each hot-spot was visited once and before starting an interview, potential respondent was asked to confirm that he had not been interviewed before for this project.

Before the commencement of each interview, the aim of the study was explained to potential candidates. Written informed consent was obtained from all participants. The participants were asked to sign or mark the consent form to indicate their consent. No attempt was made to validate the signature against any formal identification; the signature or mark was not scanned or otherwise captured in the electronic database; and the paper versions of the consent forms and questionnaires were secured in locked storage after data entry. A copy of the informed consent form was provided to each individual, which included contact information for the researchers.

### Data management

Data from the questionnaires were digitalized with a data entry interface using LSD software (Sistemas Integrales, Santiago de Chile, Chile) previously used for complex surveys. As a way of guaranteeing data quality – specifically, conformity to information in the printed questionnaires – all surveys were entered twice, and inconsistencies in the first digitalization were revised against the printed forms and corrected. Variables included in the analysis were reviewed to eliminate outlier values. The resulting database was transferred to STATA 8.0 for analysis.

### Variables & statistical analysis

Descriptive statistics were generated for the socio-demographic characteristics of MSM and reported rates of condom use. To examine associations between condom use with last each of the 3 partner and socio-demographic variables, logistic regression models were estimated, with condom use with each of the 3 last partners as a dependent variable, using an encounter panel allowing up to 3 observations per MSM. A significance level of 95% was applied for all analyses.

For descriptive statistics as well as regression, the design effect of cluster sampling was taken into account to estimate robust errors. The multivariable model applied to identify association with condom use was estimated using town fixed effects and random effects by MSM.

To facilitate inclusion in the multivariable model and interpretation of estimates, continuous variables were categorized into age, years of sexual life, years of schooling, and price paid for condoms. In each case, between three and four groups were created, according to the observed variable distribution. Additionally, three indices were generated and included in the model, specifically, socio-economic level, social capital, and life skills.

A socio-economic index was created using a polyserial correlation model with net income, possessions, and level of education as principal components. The resulting scores obtained with the principal components methodology were categorized in quartiles.

The social capital indicator was established using variables indicative of the MSM perception of the support they could receive in specific situations, while the life skills indicator was generated by taking into consideration variables focusing on self-perception of empowerment for condom use. The methods used for these two indices were analogous to those applied for a similar study in India [11].

All variables were introduced simultaneously in the model. Interactions between variables were explored, in particular those between social capital and life-skill indexes, and socioeconomic level and condom price categories.

Since it is argued that in the interpretation of odds ratios there is a tendency to over-estimate the risk (or protection) attributed to a variable [12], prevalence ratios were estimated indirectly from odds ratios according to a formula developed by Zhang and Yu [13]. While a number of alternative direct methods may be employed to assess prevalence ratios, most do not allow the estimation of two-level clustering.

The outcome variable for multivariate analysis was condom use, defined as whether condoms were used during intercourse with a given partner, that is condom use with each of the last 3 times he had penetrative sex (regardless of whether the last three times were with three different partners, two different partners or a single partner. Data were collected for up to 3 sexual encounters (last, penultimate, and antepenultimate).

## Results

Overall, 2,594 MSM from eight cities were interviewed, representing about 63% of those reported in the mapping (4,258). Because the design of the evaluation, sampling in the larger cities (Quito & Guayaquil) was a lower proportion of the mapped MSM (29%), while in the other cities the number of MSM interviewed was larger than those identified by the mapping.

Table 1 shows the percentage distribution of participants, according to self-identification by sexual identity (this category results from focus groups with members of the population). Categories were derived from the qualitative work undertaken to refine the survey instruments.

Table 2 depicts an overview of the characteristics of MSM participating in the survey. It is a young population (average age of 25 years, CI95% between 22 and 27 years), and mostly single (78%), although a quarter reported having at least one child.

Regarding education levels, in addition to a high level of literacy (94%), the average years of schooling (9.7 years; 95% CI 8–11) reveals a population with more than primary education, with a large proportion of individuals completing higher-level studies.

Only 10% of the MSM interviewed were involved in sex work (i.e., sold sex). The majority worked in other fields, lived with their families (consistent with the reported age), and did not engage in remunerated activities.

In terms of openness about sexual preferences, the observed tendency was greater awareness in the community than family. Thus, while 87% reported that the community was aware of their sexual preferences, only 29% assumed that their family knew.

Despite the reportedly high community awareness of sexual preferences, which may be interpreted as a sign of acceptance, 25% confirmed aggression from the police due to their sexual identity.

Although the average number of partners per week is small (an average of 1), 25% of interviewees reported they had never used a condom. Only 16% were aware of where free condoms could be obtained.

Taking into account the last three sexual partners of each man interviewed, only 25% used a condom consistently with all three partners, and 55% never used a condom. Finally, 30% of the most recent sexual partners were women.

### Correlates of condom use

Bivariate and multivariate associations with condom use at each of last 3 partners are shown in Table 3. Robust confidence intervals were estimated taking into account both intra-cluster and intra-individual correlations.

The prevalence ratio for condom use among unmarried in relation to married subjects was 5.45 (95% CI 4.236–6.37), and 0.9 for a female partner (95% CI 0.81 – 0.99). Data on age and education obtained from multivariate or bivariate analyses were not statistically significant.

The reported condom price was strongly correlated with condom use, suggesting that a higher cost significantly reduces use. It is important to highlight that this occurs in a model including the socio-economic level, and thus the issue is not the absolute cost of the condom, but rather its relative price.

MSM who reported a higher number of partners in the preceding week (five or more) used a condom more often (RP 1.06; 95% CI 1.05 – 1.07), although the effect was minor.

Alcohol consumption was not associated with condom use, either positively or negatively, and neither was family awareness of sexual preference.

MSM are more likely to use a condom with new partners than those they already know. Life skills also correlated strongly with condom use. However, the social capital indicator employed is not associated with the use of condoms.

Finally, the socio-economic level plays an important role, and is directly proportional to the use of condoms. In this case, a dosage effect is not clear, but MSM belonging to the higher half of the socio-economic divide use condoms more often. Significantly, this result was obtained by controlling for education level and condom price.

## Discussion

The results presented reveal a low proportion of protected sexual intercourse in a large sample of Ecuadorian MSM, confirming that these groups are highly vulnerable in terms of STI and HIV. In fact, almost one-quarter of the participants reported that they had never used a condom. More than half the participants had unprotected sex with their last 3 partners, while only a quarter used a condom with all 3 partners. These data are consistent with other studies on MSM in the region, which reveal low proportions of condom use.

In terms of characteristics associated with condom use, a significant correlation was evident with the socioeconomic level, but not years of schooling. The data suggest that the effect of the socioeconomic level has more to do with wealth and less with education. This is reinforced by the observed association with condom price. Specifically, access to condoms is limited by economic capacity. While it is important to investigate why this happens even among higher socio-economic strata, an initial strategy should focus on increasing access to free condoms.

Similarly, the relationship between socio-economic level and condom use indicates the need for more comprehensive prevention strategies. Other studies have established a correlation between economic limitations among MSM and HIV infection [14].

We additionally observed a strong association between life skills (a vast concept, focused in this case on empowerment for condom use) index and condom use. Life skills improvements require enhanced self-esteem, which is potentially one path for preventive interventions. Further research into the effects of social capital is required.

Another important aspect is the potential bridge between MSM (the population with the highest HIV prevalence in Ecuador) and the general population by means of bisexual contact. Condom use is lower among those who report being married to a woman, and even more so when the sexual partner is a woman. Given the biological susceptibility of women to STDs, this behavior could spread infection outside MSM communities.

The HIV/AIDS epidemic in Ecuador is largely confined to the MSM population, as is the case in most Latin American countries. Nevertheless, recent years have seen a trend towards feminization of the epidemic that may be related to a high prevalence of bisexual MSM in the region, as illustrated in the results of this survey [15].

### MSM in Ecuador

The population included in the MSM category is a very heterogeneous group. The width of the confidence intervals reflects important differences with regard to city, which need to be integrated in the design of interventions. For example, the percentage of MSM who had never used a condom varied from 4% to 38%, depending on the city.

The participants of the survey turned out to be a young and reasonably well-educated population, with an average of almost 10 years of schooling. Consistent with the profile of the group in the region, bisexuality was very common. In fact, a fifth of participants were married, and almost a quarter were parents. This specific profile needs to be considered for prevention, since messages focusing on men having sex exclusively with other men may not be adequate to target this population. Importantly, meeting places identified in this survey provide an avenue for reaching a broad spectrum of MSM, who are important channels for communicating information. The fact that more than 90% of this heterogeneous population is literate also provides an opportunity to use more complex and targeted messages.

While the interpretation of "community" is currently ambiguous, 87% of participants disclosed that their sexual preference was known in the community, while only 29% reported the same with respect to their family. This suggests a double life, which also has implications for prevention. It is important to distinguish prevention messages in environments where these individuals are in their MSM life from those in their heterosexual life. It also highlights the importance of providing information within the context of an environment that can be hostile to same-sex sexual behaviour.

It is important to acknowledge the limitations of these results, which are due, in part, to the type of sample as well as the analysis performed. Firstly, although every effort was made to include a representative sample of all MSM in the cities, it was, at best, a model of the most accessible MSM at the moment of the survey. Since we performed a cross-sectional analysis, causality was not attributed to proposed independent variables. Although we believe it unlikely that some men were interviewed twice (if they told the interviewer that they had already responded to the survey then the interview was aborted and there were no financial incentives for participating), no systematic effort was made to exclude repeat interviews.

With any stigmatized population there are concerns about how potential stigma and discrimination may have introduced participation bias. Despite assurances of anonymity, potential respondents who are more worried about possible breaches of confidentiality may have been less likely to participate in the survey. It is difficult to speculate about the likely direction of such bias. On the one hand it may be that those most worried about disclosure are men who lead heterosexual lives and rarely venture into MSM venues. Or, one could argue that men at highest risk, those selling sex or using drugs, may be most wary of law enforcement and may be less likely to participate in the survey.

## Conclusion

The low levels of condom use in a high HIV-prevalence population necessitate urgent and immediate action. Preventive interventions, including revision of mechanisms to promote condom use that do not seem to be working, must be a priority in this population. MSM is the population group in Latin America most affected by this epidemic, and yet public health actions have been neither appropriate nor sufficient [1]. The results reported here for Ecuador are possibly representative of other countries in Latin America. In this sense, it is imperative that priorities and prevention strategies be re-evaluated for effective response to this epidemic.

## Competing interests

The author(s) declare that they have no competing interests.

## Authors' contributions

JPG conceived the idea of this report, contributed to the design of the study, designed and led the analysis and interpretation of data, and drafted the manuscript. DM, KM & FS contributed to the idea of the report and study design, and assisted in drafting of this manuscript. SMB led the study design, and contributed to the idea, as well as the design, interpretation and drafting of this report. All the above authors read, commented on, and approved the final version of the document.

## Pre-publication history

The pre-publication history for this paper can be accessed here:



## Figures and Tables

**Table 1:** Percentage of MSM by category of self-identification (n = 2,594)

**Type**	**Number (percentage)**
Active bisexual	906 (35%)
Active and passive bisexual	108 (4%)
Passive bisexual	57 (2%)
Masculine gay	571 (22%)
Heterosexual	350 (13%)
Transsexual	18 (1%)
*Drag-queen*	48 (2%)
Transgender	43 (2%)
*Transvestite*	*310 (12%)*
*SW Cachero*	*116 (4%)*
Other	67 (3%)

**Table 2:** Socio-demographic characteristics of participating Ecuadorian MSM (n = 2,594)

**Characteristic**	**Mean (95% CI)**
Age (mean & 95% CI*)	24.8 (22.3 – 27.3)
Unmarried (percent & 95% CI*)	78% (72% – 84%)
Has at least one child (percent & 95% CI*)	25% (18% – 32%)
Can read and write a message/letter (percent & 95% CI*)	94% (91% – 97%)
Years of schooling (mean & 95% CI*)	9.7 (8.4 – 10.9)
Sex work as a main income source (percent & 95% CI*)	10% (6% – 14%)
Family is aware of his sex preference (percent & 95% CI*)	29% (21% – 38%)
Community is aware of his sex preference (percent & 95% CI*)	87% (79% – 95%)
Has been a victim of police aggression because of his preference (percent & 95% CI*)	25% (17% – 34%)
Has never used a condom (percent & 95% CI*)	24% (12% – 36%)
Knows where to obtain a free condom (percent & 95% CI*)	16% (12% – 21%)
Average number of male partners in the preceding week (median, IQR)	1 (0 – 5)
Used condom with all 3 last partners^# ^(percent & 95% CI*)	25% (15% – 35%)
Not used condom with any of 3 last partners^# ^(percent & 95% CI*)	55% (45% – 67%)
Last sexual partner was female (percent & 95% CI*)	30% (10% – 49%)

*Confidence intervals consider the design effect for clustering. ^# ^For some MSM, only 2 sexual partners were reported

**Table 3:** Association of selected variables with condom use for anal/vaginal sex by MSM with their sexual partners in bivariate & multivariate analysis (prevalence ratios* & 95% confidence intervals**).

**Variable**	**Category**	**Bivariate PR (95% CI)**	**Multivariate PR (95% CI)**
Female partner	No	1.0	1.0
	**Yes**	**0.78 (0.68 – 0.87)**	**0.9 (0.81 – 0.99)**

Unmarried	No	1.0	1.0
	**Yes**	**6.91 (6.18 – 7.32)**	**5.45 (4.26 – 6.37)**

Schooling years	Up to 5 years	1.0	1.0
	6 to 8 years	1.01 (0.83 – 1.12)	0.97 (0.78 – 1.11)
	9 years and over	1.09 (0.66 – 1.65)	0.89 (0.46 – 1.52)

Age (years)	Under 19	1.0	1.0
	19 to 22	1.08 (0.94 – 1.17)	1.11 (0.98 – 1.2)
	23 to 28	1.11 (0.98 – 1.21)	0.95 (0.76 – 1.09)
	29 and over	0.96 (0.74 – 1.14)	0.93 (0.71 – 1.1)

Price of condom	Low	1.0	1.0
	**Medium**	**0.08 (0.05 – 0.12)**	**0.09 (0.05 – 0.13)**
	**High**	**0.27 (0.18 – 0.4)**	**0.60 (0.42 – 0.81)**

Number of partners in previous week	None	1.0	1.0
	1	**1.14 (1.02 – 1.23)**	1.03 (0.88 – 1.15)
	2 a 4	**1.3 (1.25 – 1.32)**	1.08 (0.94 – 1.18)
	**5 and over**	**1.07 (1.06 – 1.07)**	**1.06 (1.05 – 1.07)**

Family is aware of sexual preference	No	1.0	1.0
	Yes	**1.27 (1.16 – 1.36)**	1.15 (1 – 1.27)

Respondent consumed alcohol prior to sexual activity	No	1.0	1.0
	Yes	1.03 (0.97 – 1.09)	0.99 (0.91 – 1.05)

Sexual partner was a new one	No	1.0	1.0
	**Yes**	**1.29 (1.23 – 1.35)**	**1.29 (1.22 – 1.35)**

Life-skills index	Low	1.0	1.0
	**Medium**	**1.38 (1.3 – 1.43)**	**1.41 (1.33 – 1.45)**
	**High**	**1.85 (1.83 – 1.86)**	**1.84 (1.79 – 1.86)**

Social capital index	Low	1.0	1.0
	High	1 (0.7 – 1.36)	1.28 (0.77 – 1.87)

Interaction life-skills index & social capital index	Low and low		1.0
	Medium and high		0.77 (0.45 – 1.03)
	High and high		0.62 (0.28 – 1.04)

Socio-economic level index	Low	1.0	1.0
	Medium low	0.97 (0.82 – 1.09)	1.02 (0.83 – 1.15)
	**Medium high**	1.04 (0.84 – 1.18)	**1.23 (1.12 – 1.3)**
	**High**	1.06 (0.85 – 1.22)	**1.2 (1.01 – 1.31)**

*Calculated by using the formula RR = OR/[(1-P_0_)+(P_0_*OR)]. [8] **95% confidence intervals were estimated considering fixed effects by city and random effects by MSM
